# Counterfeit Drug Penetration into Global Legitimate Medicine Supply Chains: A Global Assessment

**DOI:** 10.4269/ajtmh.14-0389

**Published:** 2015-06-03

**Authors:** Tim K. Mackey, Bryan A. Liang, Peter York, Thomas Kubic

**Affiliations:** Department of Anesthesiology, University of California, San Diego School of Medicine, San Diego, California; Division of Global Public Health, University of California, San Diego School of Medicine, Department of Medicine, San Diego, California; Global Health Policy Institute, San Diego, California; Pharmaceutical Security Institute, Vienna, Virginia

## Abstract

Counterfeit medicines are a global public health risk. We assess counterfeit reports involving the legitimate supply chain using 2009–2011 data from the Pharmaceutical Security Institute Counterfeit Incident System (PSI CIS) database that uses both open and nonpublic data sources. Of the 1,510 identified CIS reports involving counterfeits, 27.6% reported China as the source country of the incident/detection. Further, 51.3% were reported as counterfeit but the specific counterfeit subcategory was not known or verifiable. The most prevalent therapeutic category was anti-infectives (21.1%) with most reports originating from health-related government agencies. Geographically, Asian and Latin American regions and, economically, middle-income markets were most represented. A total of 127 (64.8%) of a total of 196 countries had no legitimate supply chain CIS counterfeit reports. Improvements in surveillance, including detection of security breaches, data collection, analysis, and dissemination are urgently needed to address public health needs to combat the global counterfeit medicines trade.

## Introduction

One of the most complex and challenging problems faced as a result of the globalization of health-care delivery is securing the integrity and safety of the global medicines supply chain. Dangerous forms of pharmaceuticals are illicitly sold by criminal elements and illegal transnational organizations creating patient safety and public health dangers that undermine public and private investments in health care.^1^–^3^ The scope of this illegal international trade is broad and complex, includes products spanning a host of therapeutic classes and lifesaving treatments, involves multiple actors and enabling stakeholders, and impacts global populations from the poorest to wealthiest.^1^–^6^

Yet, despite recognized dangers to global public health, reports compiled by public and private stakeholders (including law enforcement, regulatory agencies, manufacturers, and customs officials) show that the scope, production, distribution and sales of substandard, spurious, falsely labeled, falsified, counterfeit medicines (SSFFC) continues to increase.^2^,^7^,^8^ For example, the diversion/theft of pharmaceuticals increased 66% while counterfeiting incidents during this same period increased 122% according to publicly available data for the period from 2005 to 2010 from the Pharmaceutical Security Institute (PSI), a not-for-profit, membership organization of pharmaceutical company security directors.^8^ PSI currently has the membership of 28 major pharmaceutical companies (Table 1). Reports from other international and professional organizations including the United Nations (UN) Office of Drugs and Crime, World Health Organization (WHO), Institute of Medicine (IOM), and the National Association of Boards of Pharmacy, have also detailed increasing challenges to safeguarding the global medicine supply chain, yet exact figures on the true scope of the problem remain elusive.^4^,^5^,^9^–^11^

It is also important to note the diversity of terminology generally used for the problem of “poor quality” drugs that has caused confusion and hampered progress in defining and controlling this public health problem. This includes the all-inclusive “SSFFC” terminology currently used by WHO, which lacks consensus but generally includes the key terms: “substandard” (those that fail to meet established quality specifications due to unintentional or negligent errors), “falsified” (when there is a deliberate and intentional fraud regarding the quality specification), and “counterfeit” (when there is a false representation as regards to identity/source that can include falsified/fake medicine that illegally breaches the drug supply chain).^5^,^6^

For the purposes of this study, we use the term “Counterfeit” and a report of a “Counterfeit Incident,” consistent with the definition used by PSI (which is consistent with the 1992 WHO definition of “counterfeit” medicines), defined as: a report of a medicine that was deliberately and fraudulently mislabeled with respect to identity and/or sources to make it appear to be a genuine product, whether branded or generic.^6^

## Legitimate Supply Chain

Within this transnational criminal enterprise of trade and distribution of SSFFC, a key systemic category exists: SSFFC medicines that have been detected in the legitimate or controlled drug supply chain. Specifically, we define the legitimate supply chain as “any supply chain that is either regulated/licensed by a ministry of health or other regulatory body [or] any supply chain where a patient would reasonably expect to obtain authentic product, supplied via a controlled supply chain, from the manufacturer of the product to the point of dispensing.”^12^ This definition stands in stark contrast to distribution and access to counterfeit medicines in the illicit/uncontrolled supply chain (such as unlicensed sources including establishments in the informal economy, night markets, bodegas, the Internet).

Penetration of counterfeit medicines into the legitimate supply chain is uniquely important and requires focused public health and policy research for three key reasons. First, these incidents have not been well evaluated in any systemic manner, compared with work on illegal supply chains and unregulated markets.^4^,^13^–^16^ Second, counterfeit medicines have already been documented as penetrating the legitimate supply chains of weak and highly controlled drug supply chains alike (such as counterfeit antimalarials in community pharmacies and counterfeit anticancer drugs being detected in the U.S. legitimate supply chain), indicating the need for further research to address existing vulnerabilities and supply chain complexities that are contributing factors for counterfeit penetration.^15^–^18^ Finally, this assessment supports the policy work of governments in providing fact-based recommendations to key opinion leaders and policymakers in making necessary changes to surveillance, security, and improvements to pharmaceutical governance of domestic, regional, and international drug supply chains to prevent future Counterfeit Incidents.

Hence, we were interested in reviewing data describing penetrations of counterfeit medicines in the legitimate supply chain. To accomplish this objective, we reviewed data made available to us and collated from multiple data sources by PSI composed of reports of Counterfeit Incidents detected in the legitimate supply chain over a 3-year period. This analysis, the first of its kind as of our knowledge, can provide insights into future development of counterfeit medicines surveillance and data collection efforts, as well as inform international policy efforts to promote medicine supply chain security.

## Materials and Methods

Our general approach was to review information collected in the proprietary PSI Counterfeit Incident System (CIS) database limited to instances of legitimate supply chain penetration for the reporting period of 2009–2011,† †Counterfeit Incident reports in CIS did not include normalized data on the level of supply chain penetration where counterfeit medicines were detected. However, we note based on the limited data available that detection often occurred with pharmacies, distributors/wholesalers, hospitals, and warehouses. employing a cross-sectional methodology. We focused on descriptive data analysis and assessment, since CIS data is not normalized and relies on nonrandom reporting from multiple data sources that may not always be standardized at the time of reporting. This may include oversampling due to under or over reporting of Counterfeit Incidents from different international jurisdictions and varying information sources, making analysis difficult. Hence, our goal was to evaluate the CIS database for reported Counterfeit Incidents in the legitimate supply chain as a potential means to identify macro counterfeit drug characteristics and to inform future development of Counterfeit Incident surveillance, reporting, and data analysis and use.

We reviewed data from the CIS database at the PSI Vienna, VA, headquarters limited to Counterfeit Incidents reported in the legitimate drug supply chain. Data were also redacted by PSI to blind any information on specific medicine formulation (i.e., brand or manufacturer). We then used this parsed data to assess Counterfeit Incidents by therapeutic class, route of administration, and reporting source. Finally, we also assessed the economic and governance characteristics related to countries reporting at least one Counterfeit Incidents in the CIS, including factors: country income (source: World Bank), geographic region (source: UN and WHO), and perception of corruption (source: Transparency International [TI]).

Specifically, we used the CIS because it is the only dynamic, functional global database that secures and collates information on Counterfeit Incidents, illegal pharmaceutical diversion, and theft worldwide. It is also arguably the most inclusive, being the only database globally receiving and analyzing reports from open, publicly available sources, closed PSI member company reports, national regulatory and public health agencies, as well as public–private partnerships, and validates these reports using law enforcement–based analytic techniques (including verification of open- and closed-sourced reports using multilingual analysts). However, it should be noted that data from CIS is proprietary to its members and not made available to the public other than in yearly aggregate trend reporting on PSI's website. Hence, though the specific data we received for analysis is not publicly available, aggregate CIS data has the potential to inform PSI members, drug regulators, national health systems, and law enforcement officials about the macro-level trends and dangers of counterfeit medicines. In addition, CIS information collated in PSI's annual situation report available to PSI members and certain stakeholders also provide important information on how to address future security breaches and the identification of criminals involved in the trade.

We first assessed reports of Counterfeit Incidents based on the definition of “counterfeit” used by PSI and as previously mentioned. This definition includes medicines where there is no active pharmaceutical ingredient (API), undeclared or unapproved API in a medicine, or insufficient API, all issues that pose clear patient safety risks. The type of Counterfeit Incident is further divided into seven distinct CIS Counterfeit Incident subcategories including: undeclared API (i.e., product where API was present but undeclared); counterfeit API (CF API, i.e., product where an unapproved API was present); counterfeit product only (CF product only, i.e., actual pharmaceutical product [e.g., pill] was counterfeit but no assessment of packaging); mimicked product (i.e., unapproved product with trademark violation); counterfeit packaging only (CF packaging only, i.e., packaging was counterfeit but no assessment of actual pharmaceutical product); counterfeit product and packaging (CF product and packaging); and counterfeit-unclassified (i.e., the product is reported as counterfeit but specific category not known or verifiable).

A single Counterfeit Incident can involve multiple drugs, multiple drugs in the same therapeutic category, as well as multiple drugs within the same therapeutic category but in different formulations. Unfortunately, reporting of counterfeit drug quantities is not currently a normalized or a required field in CIS. In addition, it is often difficult for drug regulators and law enforcement officials to accurately ascertain the quantities of “counterfeit” medicines without separate validation (such as the use of mass spectrometry or liquid chromatography) that may not be pursued. We note however, that based on the limited data on estimated quantities contained in CIS, quantities generally average in the range of the hundreds to thousands, with the exception of large counterfeit drug seizures.

We assessed the therapeutic class of drugs reported in Counterfeit Incidents in two ways. First, we used PSI's 16 therapeutic class divisions (alimentary, anti-infectives, blood agents, cardiovascular, central nervous system, cytostatics, dermatological, genitourinary, hormones, hospital solutions, metabolism, musculoskeletal, not available [NA], respiratory, other parasitology, and sensory organs) and then reported descriptive frequencies for each.‡ ‡The 16 therapeutic categories listed are the standard categories reported and collected by the CIS and are explained in further detail in Table 1. However, there may be certain subcategories of the standard therapeutic classes that warrant further study including drugs that treat psychological conditions (included in “Central Nervous System” category) and antimalarials that have been widely detected as counterfeited (included in the “Anti-Infective” and also certain treatments in the “Parasitic” category). Second, we performed cross tabulation analysis to observe any associations between therapeutic class and counterfeit categories.^19^ We also assessed the route of administration (oral pill, injectable, inhalable, topical, eye drops) of drugs reported in Counterfeit Incidents.

With regard to reporting sources, we used the CIS six specific categories of information sources. Broadly these include both external (non-PSI members) and internal (PSI pharmaceutical manufacturer member or partner) information sources. These specific categories are External Healthcare Agency (a generic term for health-related government agencies such as drug regulatory agencies, ministries of health); External-Other (undefined nonmember reports generally from open sources such as the media); External Industry Source (non-PSI member pharmaceutical manufacturer/distributor); External Law Enforcement; Retailer (external point-of-sale source such as a community pharmacy, etc.); and Internal (i.e., PSI member).

For economic and geographic analysis, we used three sets of data for income level and geographic region. The first was the World Bank data by country income.^20^ This is an income-based categorization of countries including low income, lower middle income, higher middle income, and high income. The second was the geographically oriented UN-based system of country classifications by region and subregion: Africa, Americas, Asia, Europe, and Oceania and 21 potential subregions.^21^ The third was geographically oriented categorization based on the six WHO regional offices.

Finally, we assessed countries identified in the CIS with at least one report of a Counterfeit Incident in the legitimate supply chain for relative perception of corruption using the 2012 TI Corruption Perceptions Index (CPI). We used TI CPI scores as they provide a simple and easy to use composite index (comprises a combination of polls from a variety of experts and institutions) specifically measuring the perception of public sector corruption within a given country/territory. TI is a not-for-profit, nongovernmental watchdog–based organization in Germany and represented by a Board of Directors from 12 different countries. Any country that had at least one incident report was included in this analysis. Scoring is from 0 to 100, where 0 is defined as highly corrupt, 100 as completely accountable/transparent. TI CPI Index rank is the country's rank from highly corrupt countries (toward 1) versus “clean” countries (higher numbers).^22^ It should be noted that other organizations such as the UN Development Program and the World Bank (e.g., Worldwide Governance Indicators Project) also provide indicators/measures that may also be beneficial for examining levels of public sector corruption.

## Results

Globally, there were 1,510 total reports of Counterfeit Incidents. Counterfeit Incident reports included 1,799 different counterfeit medicine detections (“Detected Counterfeit Medicines”) penetrating the legitimate medicines supply chain for the 36-month period from 2009 to 2011 as reported in CIS. Specifically, a single Counterfeit Incident reported to CIS can involve the detection of a single counterfeit medicine product or multiple counterfeit medicine products in the same incident identifier and includes information regarding the product's therapeutic class. These CIS reports came from internal PSI members, open-source reports including PSI-validated information from drug regulators, and incidents identified through liaison and joint investigative efforts with international and national police agencies, as described above.

### Detected counterfeit medicines.

The most common type of counterfeit category reported in CIS for Detected Counterfeit Medicines was the counterfeit-unclassified category (51.3%, *N* = 775), followed by CF product and packaging (43%, *N* = 649). Of the remaining categories, most garnered much fewer reports: CF packaging only (3.3%, *N* = 50), CF product only (1.3%, *N* = 19), mimicked product (0.7%, *N* = 11), CF API (0.3%, *N* = 4), undeclared API (0.1%, *N* = 2).

Therapeutic categories for the Detected Counterfeit Medicines in Counterfeit Incident reports are summarized in Table 2. The primary therapeutic drug class was anti-infectives (21.1%, *N* = 380) followed by genitourinary (14.5%), cardiovascular (11.6%), and central nervous system (11.0%), although every therapeutic category was represented. The high frequency of the anti-infective category in CIS reports is not surprising, given private and public sector observations that lifesaving products (i.e., medicines that treat or cure diseases that generally carry the threat of morbidity or mortality) including both branded and generic anti-infectives/antibiotics, such as antimalarial drugs, are frequently detected as counterfeit.^4^,^14^,^15^,^23^,^24^

Assessing the number of anti-infectives reported to the product category genitourinary (which includes erectile dysfunction [ED], e.g., “lifestyle” drugs defined as drugs used to improve the quality of life rather than for alleviating pain or curing disease^25^), anti-infectives have 1.46 times more detected reports than genitourinary drugs in the CIS database (380 or 21.1% versus 260 or 14.5%). This is notable given that noncommunicable disease, branded products are likely the focus of internal member-based CIS database reporting, yet still are found with a lower number of Detected Counterfeit Medicines reports than anti-infectives.

In addition, even considering all genitourinary counterfeits as lifestyle drugs (likely overinclusive, e.g., some prostate drugs may be included), lifesaving-related drug categories, i.e., anti-infectives (21.1%), cardiovascular (11.6%), central nervous system (11.0%), and alimentary (9.1%), nevertheless represent the majority (52.8%) of all detected counterfeit medicines reported as penetrating the legitimate supply chain in CIS reports. However, when attempting to assess any relationship between the therapeutic categories and counterfeit drug incident categories using cross tabulation analysis, the high number of counterfeit-unclassified drugs (*N* = 775) made any potential associations with other CF categories infeasible.

Since most medicines are administered orally, we expected that most of the detected counterfeit medicines in the CIS reports would have oral route of administration, which is what we observed. Greater than three-fourths (77.3%) of counterfeit medicines were oral dosage formulations. The second most reported route of administration category was injectable biologic drugs (15.4%), a formulation that has also been detected in other publicly available data reported by PSI.^26^

### Counterfeit incident and country characteristics.

**Figure 1.**
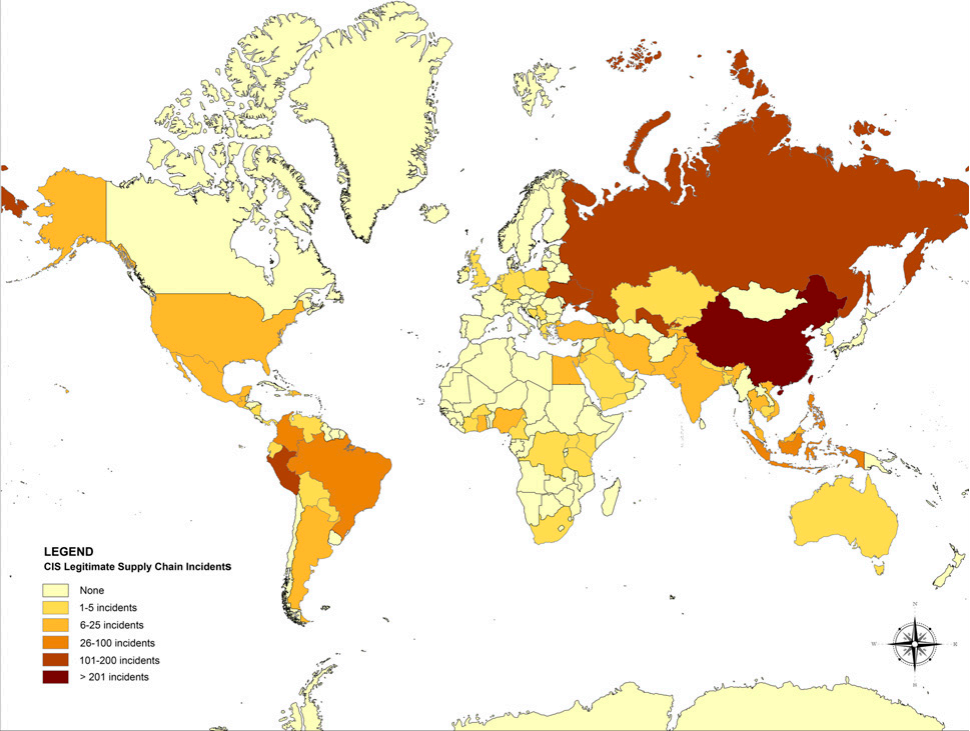
Global map of Counterfeit Incident System (CIS) Counterfeit Incidents by country.

In the geographic country analysis of CIS data, we found that China dominated as the country reporting source in CIS reports of Counterfeit Incidents, with about a quarter (27.6% *N* = 417) of all reports (*N* = 1,510) coming from this single country (Figure 1

). It should be noted that reports identifying China as the source of a Counterfeit Incident may or may not be generated by the government of China or have sufficient information to include without further investigation by PSI. In total, 69 countries were identified as reporting legitimate supply chain Counterfeit Incidents into CIS, with the top 5 countries being China (27.6%, *N* = 417, Rank #1 in global population), Peru (11.6%, *N* = 175, Rank #43), Uzbekistan (10.9%, *N* = 165, Rank #45), Russia (8.4%, *N* = 127, Rank #10), and the Ukraine (7.2%, *N* = 108, Rank #32), in total representing 65.7% (*N* = 992) of all Counterfeit Incident reports.

In addition to assessing the specific countries identified with reports of Counterfeit Incident penetrations in the legitimate supply chain, we were also interested in countries that did not report any Counterfeit Incidents into CIS. Overall, we found 127 (64.8%) out of a total of 196 countries globally did not appear in any CIS Counterfeit Incident reports of penetration of counterfeits into their legitimate supply chains within the study time frame (Table 3).

CIS reports of Counterfeit Incidents analysis also indicated External Healthcare Agencies (which includes ministries of health and drug regulatory agencies) as the top reporting information source, representing over half (58.1%, *N* = 878) of all identified Counterfeit Incidents involving legitimate supply chain penetrations. This was followed by internal reports by PSI members (15.2%, *N* = 229), External-Other (14.0%, *N* = 211), External Law Enforcement (12.1%, *N* = 183), External Industry Source (0.5%, *N* = 8), and retailer (0.1%, *N* = 1). The level of counterfeit-unclassified and CF product and packaging, the two largest overall counterfeit subcategories in these reports, is similar across public and private/PSI member information sources (Table 4).

Assessing country income using the World Bank income divisions, middle income countries dominate Counterfeit Incident reporting (Table 5). Upper and lower middle income countries comprise 93% (*N* = 911 + 501 = 1,412/1,510) of all CIS Counterfeit Incident reports in the legitimate supply (noting the high number of reports from China). Countries at the extreme ends of the income spectrum appear to encompass a relatively small fraction, potentially reflecting strong infrastructures protecting supply chains in high-income countries, and limited drug supply safety and counterfeits detection systems in low-income countries. Here, External Healthcare Agency and External Law Enforcement sources comprise most of CIS reports.

When assessing regional distribution of CIS legitimate supply chain penetration reports for individual countries with at least one Counterfeit Incident reported in CIS (*N* = 69 total countries) using both UN region (Asia, Americas, Africa, Europe, and Oceania) and subregions, we found that Asia (42.0%, *N* = 29) makes up the majority of Counterfeit Incident reports. Regional distribution is then followed by the Americas (24.6%, *N* = 17), Africa (17.4%, *N* = 12), Europe (14.5%, *N* = 10), and Oceania (1.4%, *N* = 1). Using the more granular UN subregion categorization, 50.7% (*N* = 35) of all CIS reported countries are within southeast Asia (8.7%), southern Asia (8.7%), and western Asia (14.5%) as well as central America (7.2%) and South America (11.6%).

We also assessed regional distribution of CIS legitimate supply chain penetrations by the six WHO geographic office regions. This includes the regional offices for Africa (AFRO), the Americas (PAHO), the eastern Mediterranean (EMRO), Europe (EURO), southeast Asia (SEARO), and the western Pacific (WPRO). Using this geographical distribution, the highest number of reports emanated from EURO (24.6%, *N* = 17) and PAHO (21.7%, *N* = 15) (Table 6).

Finally, we assessed the potential relationship between countries with CIS reported Counterfeit Incidents and perception of relative corruption in these countries. Using the TI CPI to determine the relative index score of countries with CIS documented penetrations of their legitimate supply chain, we found that these countries are moderately corrupt, with a mean score of 39.09 (*N* = 69, standard deviation 16.41, TI CPI score range 17–85). However, these scores are not weighted for number of Counterfeit Incidents in an individual country. A TI CPI score of 50 reflects a government perceived as equally corrupt/transparent; consequently, as a group, the mean score indicates moderately corrupt perceptions of these countries but with wide variation (includes countries perceived as having high levels of corruption [score of 17] to countries perceived to have a low level of corruption [score of 85]). This may indicate there is no “typical” country in terms of corruption perception that is included within CIS legitimate supply chain penetration reports.

## Discussion

To our knowledge, this is the first description and assessment of global legitimate medicine supply chain penetration by counterfeit medicines combining public and private sources to date. It is important to note that other reports on Counterfeit Incidents are available and include national customs and crime detection work, surveys done by national and international organizations, data analysis in scientific journals, and reports such as the 2013 IOM report that also used information from CIS.^1^,^4^,^5^,^14^–^16^,^27^–^29^ However, the results of these reports and detection systems may be limited to certain specific therapeutic classes and can be excessively delayed due to political, communication, legal, and economic reasons, as well as testing inaccuracies/delays. As further preliminary work on the subject, our findings point to directions where information and study could be beneficial to combine improvement in counterfeits knowledge globally as well as promoting global public health interventions and surveillance in this area.

Three key areas emerged from analysis of CIS reports: the need for greater consistency, standardization, and robust data collection for analysis; enhanced surveillance and reporting by countries identified as not reporting into CIS, and the need to increase diversity and participation by important reporting sources in the CIS for legitimate supply chain penetration incidents. For example, certain countries with high frequency of CIS legitimate supply chain Counterfeit Incident reports may have active inspection regimes and/or better public sector reporting compared with other countries where incidents were not detected. This inhibits generalization and policy decisions based on CIS data and points to the need for more prompt multistakeholder reporting that can lead to proactive and remedial action.

Improvements to CIS and other counterfeit surveillance methods should specifically include further development and coordination of a “single point of contact” system composed of relevant country authorities that will be responsible and accountable for conducting surveillance and reporting Counterfeit Incidents to a central global system. Specific to CIS, better and normalized data regarding the verified quantity of detected counterfeit medicines and details on the level/location of detected supply chain penetrations may provide more robust information, but have their own challenges in ensuring accurate reporting based on information collected from the field. Hence, we believe future efforts should focus on cooperative harmonization to expand the diversity, sources, number, and quality of reports to improve data collection across all areas.^1^

Taking lessons from aviation (such as the U.S. Aviation Safety Reporting System or “NASA program”) and medical patient safety reporting, using simple, secure online forms with limited and standardized categories, several specific reporting forms (e.g., aviation pilot, air traffic controller, mechanical, cabin crew), drop-down responses, checkboxes, and a single field for open narrative, may be a useful foundation.^30^,^31^ Because forms are anonymized by a third-party contractor when necessary or desired before analysis and are accompanied by legal protections, reporting from a wide range of stakeholders is promoted. This would be essential in the complex system of health-care privacy and security associated with detection of counterfeit medicines in the controlled supply chain.

Specifically, the basic principles that underpin the CIS provide a possible foundational model for the development of a global SSFFC surveillance system that includes efforts by WHO to implement a new open web-based system for reporting cases of counterfeit, falsified and/or substandard medicines.^32^ CIS characteristics include independent validation of open and closed data sources; sourcing of reports from multiple stakeholders; use of specific fields for type of incident, geographic location, and product information; and the use of follow-up reports to update incidents as investigations unfold. Crucial to bringing the benefits of this approach to sustained global efforts will be harmonizing definitions and reporting fields, establishing cooperative public–private approaches to shared reporting responsibilities, and stakeholders committing to an appropriate level of transparency and public reporting of data to inform all impacted partners. Transparency that does not compromise law enforcement efforts is important to improve public awareness of the issue, disseminate important information on the inherent risk characteristics of the counterfeit medicines trade to promote prevention, and shared capacity building for prompt detection and remedial action.

In addition, we believe enhancements to the existing CIS could also improve robust data and analysis of Counterfeit Incidents in the legitimate supply chain. Some suggestions include

1. PSI creating a harmonized reporting form and secured online entry portal with PSI members allowing for necessary editing and updating of the record as information is validated.
2. Identifying other stakeholders (e.g., customs; drug regulatory authorities; nonmembers) and collaborating to create tailored forms based on the PSI member harmonized reporting form.
3. Pilot test the program by region (e.g., 1–3 UN subregions with cooperating group of diverse stakeholders).
4. Determine lessons learned and improvements for global implementation, with potential tailoring by UN region and subregion.
5. Revisit reporting structure annually or biannually with stakeholders for review.

Such an approach would provide greater consistency and statistical power for data analysis, broaden reporting by countries and other stakeholders, and create a more representative sample that would provide a greater potential for statistical assessment and identifying macro and micro trends to better promote patient safety and shared global pharmaceutical security. It could also serve to establish a diverse, robust, and multistakeholder public–private partnership source of counterfeit medicines information that would be more amenable to discussions for improving global health governance for counterfeit medicines that are ongoing within the international community.^1^,^2^,^4^,^6^,^33^

We also believe identification of specific public health–sensitive counterfeit drug categories for policymaking attention and priority setting should be used. For example, the public health consequences associated with counterfeit vaccines should be a priority in counterfeit drug surveillance.^34^,^35^ Other drugs at potential high risk for counterfeiting, including drugs subject to critical manufacturing shortages and narrow therapeutic index drugs that require precise manufacturing to be effective, emphasize the need for this concept.^36^–^39^ Collecting robust data on key, agreed on priority specific categories may be beneficial for these risk-based approaches and result in a shift in policy focus on the basis of specific, actionable information from the field.

It should also be noted that middle-income countries are critical in focusing efforts to promote global drug safety. Although better data are clearly necessary, according to a variety of measures—economic, geographic, and third-party Counterfeit Incident reporting—Middle Income countries appear to have the potential for greater counterfeit medicines production and penetration in their legitimate supply chain.^1^,^40^–^42^ As many of these countries are emerging markets, they are increasingly both consumer and producer countries of both legitimate and illicit forms of medications; however, their domestic consumption and production of medicines may be outpacing their regulatory maturation.^43^,^44^ Weaknesses in these Middle Income countries with regard to counterfeit manufacture and export impacts global patient safety because of both the legitimate pharmaceutical trade and potential for production of counterfeits for the illicit unregulated supply chain such as the Internet.^45^

A policy focused on Middle Income countries as the beginning of a broader multistakeholder global health governance scheme could be explored. This could include cooperation between regulatory authorities, law enforcement, customs, PSI member companies, nonmember companies, academic institutions, and other stakeholders for development of a “graded best practices” set of guidelines supporting ongoing training, capacity building, technical assistance, data sharing, and partnership tailored for appropriate country development level similar to the framework established by the WHO's Good Governance for Medicines Program.^33^ This process could include

1. Creating a Best Practices Regulatory Guide for development of a counterfeit medicines public–private partnership model that engages stakeholders from health-related government agencies, manufacturers, customs and law enforcement, researchers, and civil society, focusing on development of regulatory infrastructure, surveillance, detection technology, and education focused on global patient safety starting with 1–2 cooperative middle-income countries.
2. Using PSI member expertise, developing graded best practices infrastructure guideline for improving pharmaceutical security and governance using income/development categories for use by regulatory, public health, and law enforcement agencies.
3. Providing technical assistance on possibility of vulnerabilities to pharmaceutical supply chain infrastructures, with recommended necessary components for each (e.g., lower middle income country may require information on creating a drug regulatory authority; high middle income country may require assistance on developing and implementing “track-and-trace” systems/policies that have the ability to electronically track a medicine through the supply chain and ensure its authenticity and pedigree).
4. Ensuring that reporting information in harmonized fashion is used as part of Best Practices Regulatory Guide.
5. Establishing an annual meeting for discussion, iteration, improvement, and expansion to other Middle Income markets, and subsequent development of additional tailored guidelines.

Using this approach, standardized, empirically informed and context-driven strategies can be created that can result in better infrastructures that can drive increased reporting and patient safety protections against counterfeit medicines in these potentially higher risk/higher vulnerability settings.

## Limitations

Our study has certain limitations. Authors Tim K. Mackey and Bryan A. Liang were only provided access to CIS data specific to counterfeit medicines/incidents detected in the legitimate supply chain. Hence, we were unable to make broader comparisons to data describing all counterfeit medicine incidents (including legitimate and illegal supply chains) detected in CIS. In addition, the majority of Detected Counterfeit Medicines in the sample were classified as “Counterfeit-Unclassified” indicating that though the product is reported to be counterfeit, the specific category is not known or verifiable. The high frequency of this unverified category makes it difficult to generalize any information regarding the makeup of the types of counterfeit categories detected in the legitimate supply chain. Analysis of information is also limited by oversampling of Counterfeit Incidents from a few countries, primarily China, which comprised 27.6% of the entire data set. Further complicating analysis are the 64.8% of all global jurisdictions failing to report any Counterfeit Incidents to CIS. Hence, the dominance of the unclassified/unverified counterfeit category in Detected Counterfeit Medicines and high frequency of certain countries in Counterfeit Incident reporting has the potential to skew any potential analysis and conclusions (including counterfeit category, therapeutic class, and source of information) regarding the characteristics of global counterfeit penetrations into the legitimate supply chain. We believe this reifies the need to adopt global governance and surveillance approaches to target strategies against counterfeits in the legitimate medicine supply chain.

## Conclusion

In some regions of the world, the trafficking of counterfeit medicines are crimes of opportunism, and in others, a part of a complex and organized global criminal enterprise.^46^,^47^ Effectively addressing it must combine both law enforcement and public health perspectives as well as public and private resources, for no single entity can accomplish this goal alone. As the Organisation for Economic Co-operation and Development (OECD) has noted, governments, business, and other interested stakeholders must continue to collect and assess information essential to effectively fight counterfeiting, and better information is crucial for substantive analyses to be carried out, and informed and effective policies to be put into place.^48^ We echo the OECD in calling for more cooperation as a basis for substantive action, and see the issue as one driven by the central goal of a safe global medicine supply chain. Parties with an interest in this goal must coalesce to ensure safe, effective medicines that are accessible to global populations and never compromised in the global health delivery system by supporting the development and investment in a robust and harmonized multistakeholder global counterfeit medicines surveillance system.

## Figures and Tables

**Table 1:** List of PSI pharmaceutical company members

Abbott Laboratories
AbbVie
Amgen
Astellas Pharma
Astrazeneca PLC
Biogen Idec
Boehringer Ingelheim
Bristol-Myers Squibb
Celgene
Eisai Co.
Eli Lilly and Co.
Forest Laboratories
Genentech
Gilead Sciences
GlaxoSmithKline PLC
Hoffmann-La Roche Ltd.
Johnson and Johnson
H. Lundbeck A/S
Merck and Co., Inc.
Merck KGaA
Novartis International AG
Novo Nordisk
Otsuka Pharmaceutical
Pfizer, Inc.
Purdue Pharma LLC
Sanofi-Aventis
Laboratories Servier
Takeda Pharmaceutical Co.

PSI = Pharmaceutical Security Institute.

**Table 2:** Legitimate supply chain counterfeit penetration by therapeutic category

Therapeutic class	Frequency	Percent (%)	Cumulative percent (%)
Anti-infectives	380	21.1	21.1
Genitourinary	260	14.5	35.6
Cardiovascular	208	11.6	47.1
Central nervous system	197	11.0	58.1
Alimentary	164	9.1	67.2
Musculoskeletal	146	8.1	75.3
Metabolism	138	7.7	83.0
Respiratory	68	3.8	86.8
Other (unclassified)	61	3.4	90.2
Cytostatics	58	3.2	93.4
Hormones	38	2.1	95.5
Dermatological	36	2.0	97.5
Blood agents	34	1.9	99.4
Sensory organs	5	0.3	99.7
Parasitology	3	0.2	99.8
Not reported	2	0.1	99.9
Hospital solutions	1	0.1	100.0
Total	1,799	100.0	−

According to the PSI CIS system, the term “counterfeit” refers to a report of a medicine that was deliberately and fraudulently produced and/or mislabeled with respect to identity and/or sources to make it appear to be a genuine product, whether branded or generic.

Therapeutic class definitions used by PSI CIS: Anti-infectives = drugs used to treat diseases caused by infectious agents including antimicrobial drugs, antivirals, antifungals, and antiproatozoans; Genitourinary = drugs used to treat diseases of the genitourinary or urogenital system including reproductive organs and the urinary tract; Cardiovascular = drugs that treat cardiovascular diseases and conditions impacting the heart, circulatory system, or both; Central nervous system = drugs used to treat neurological diseases of Central Nervous System, including mental health drugs; Alimentary = drugs used to treat diseases affecting the gastrointestinal tract; Musculoskeletal = drugs used to treat diseases the musculoskeletal system including anti-inflammatory and certain antirheumatic drugs; Metabolism = drugs used to treat metabolic and endocrine disorders; Respiratory = drugs used to treat respiratory diseases and infections; Cytostatics = drugs used to treat carcinomas and other nonhomeostatic proliferation of cells; Hormones = drugs used to treat diseases of hormonal imbalance, such as hormone replacement therapy; Dermatological = drugs used for the treatment of dermatologic conditions; Blood agents = drugs used to treat hematologic disorders; Sensory organs = drugs used to treat sensory and proprioception disorders; Parasitology = drugs used to specifically treat parasitic diseases; Hospital solutions = agents to disinfectant in general hospital settings.

**Table 3:** Countries with no CIS Counterfeit Incidents reports

Afghanistan	Equatorial Guinea	Macedonia	Senegal
Albania	Eritrea	Madagascar	Seychelles
Algeria	Estonia	Malawi	Sierra Leone
*Andorra*	Ethiopia	*Maldives*	Singapore
Angola	*Fiji*	Mali	Slovakia
*Antiqua and Barbuda*	Finland	Malta	Slovenia
Austria	France	*Marshall Islands*	*Solomon Islands*
Azerbaijan	Gabon	Mauritania	Somalia
Bahamas	Gambia	*Micronesia, Federated States of*	*South Sudan*
Barbados	Georgia	Moldova	Spain
Belarus	*Grenada*	Mongolia	Sri Lanka
*Belize*	Guinea	Montenegro	Sudan
Benin	Guinea-Bissau	Morocco	Suriname
Bhutan	Guyana	Mozambique	Swaziland
Botswana	Honduras	Myanmar	Sweden
Brunei	Hong Kong	Nambia	Switzerland
Bulgaria	Hungary	*Nauru*	Syria
Burundi	Iceland	New Zealand	Timor-Leste
Canada	Ireland	Niger	Togo
Cape Verde	Italy	Norway	*Tonga*
Central African Republic	Jamaica	Oman	Tunisia
Chad	Japan	*Palau*	Turkmenistan
Chile	*Kiribati*	Papua New Guinea	*Tuvalu*
Comoros	Korea (North)	Portugal	Uruguay
Congo Republic	Kosovo	Qatar	*Vanuatu*
Costa Rica	Laos	Romania	*Vatican City*
Croatia	Latvia	Rwanda	Zambia
Cuba	Lesotho	*Saint Kitts and Nevis*	Zimbabwe
Cyprus	Liberia	Saint Lucia	
Czech Republic	Libya	Saint Vincent and the Grenadines	
Denmark	*Liechtenstein*	*Samoa*	
Djibouti	Lithuania	*San Marino*	
Dominica	Luxembourg	Sao Tome and Principe	

Italic countries = not included in Transparency International Corruption Index (TI CPI).

**Table 4:** Cross tabulation table of counterfeit category by source of information

CFT subcategory	Source of information						Total (% of total incidents)
	External-other	External healthcare agency	External industry source	External law enforcement	Internal	Retailer	
CF API	0 (0%)	4 (100%)	0 (0%)	0 (0%)	0 (0%)	0 (0%)	4 (< 1%)
CF packaging only	10 (20%)	17 (34%)	1 (2%)	9 (18%)	13 (26%)	0 (0%)	50 (3%)
CF product and packaging	110 (17%)	373 (57%)	2 (< 1%)	81 (12%)	82 (13%)	1 (< 1%)	649 (43%)
CF product only	7 (37%)	7 (37%)	1 (5%)	2 (11%)	2 (11%)	0 (0%)	19 (1%)
CF- unclassified	83 (11%)	472 (61%)	4 (< 1%)	84 (11%)	132 (17%)	0 (0%)	775 (51%)
Mimic product	1 (9%)	3 (27%)	0 (0%)	7 (64%)	0 (%)	0 (0%)	11 (< 1%)
Undeclared API	0 (0%)	2 (100%)	0 (0%)	0 (0%)	0 (0%)	0 (0%)	2 (< 1%)
Total incidents	211 (14%)	878 (58%)	8 (< 1%)	183 (12%)	229 (15%)	1 (< 1%)	1,510

CF = counterfeit; CF API = counterfeit active pharmaceutical ingredient; CF packaging only = counterfeit product packaging only; CF product and packaging = counterfeit product and packaging; CF product only = counterfeit product but no assessment on packaging; CF-unclassified = product is reported as counterfeit by reporting source, but specific category not known or verifiable; Mimic product = unapproved product with trademark violation; Undeclared API = product where active pharmaceutical ingredient was present by undeclared.

CF-unclassified category represents 51% of all reports and External Healthcare Agency represents 58% of total sources of PSI CIS information reporting.

**Table 5:** Cross tabulation table of World Bank Country Income Group by source of information

Country income group	Source of information						Total (% of total Incidents)
	External-other	External healthcare agency	External industry source	External law enforcement	Internal	Retailer	
Upper middle income	154 (17%)	532 (58%)	4 (< 1%)	121 (%)	100 (13%)	0 (0%)	911 (60%)
Lower middle income	42 (8%)	318 (63%)	3 (< 1%)	35 (7%)	102 (20%)	1 (< 1%)	501 (33%)
Low income	5 (13%)	16 (42%)	0 (0%)	8 (21%)	9 (24%)	0 (0%)	38 (3%)
High income	9 (25%)	12 (33%)	1 (3%)	9 (25%)	5 (14%)	0 (0%)	36 (2%)
WB unclassified	1 (4%)	0 (0%)	0 (0%)	10 (42%)	13 (54%)	0 (0%)	24 (2%)
Total incidents		211 (14%)	8 (< 1%)	183 (12%)	229 (15%)	1 (< 1%)	1,510

World Bank Income groups are classified according to 2012 Gross National Income (GNI) per capita, calculated using the World Bank Atlas Method (see: http://data.worldbank.org/about/country-classifications).

High income = $12,616 or more; Upper-middle income = $4,086–$12,615; Lower-middle income = $1,036–$4,085; Low income = $1,035 or less; WB unclassified = these are countries that are identified in PSI CIS but are not included in World Bank income groups. This includes Taiwan in the data above.

Upper-middle-income category represents 60% of all reports and External Healthcare Agency represents 58% of total sources of PSI CIS information reporting.

**Table 6:** WHO regional groups by number of CIS countries with reports

	Frequency	Percent (%)	Cumulative percent (%)
AFRO (Africa)	11	15.9	15.9
PAHO (Americas)	15	21.7	37.7
EMRO (eastern Mediterranean)	10	14.5	52.2
EURO (Europe)	17	24.6	76.8
SEARO (southeast Asia)	8	11.6	88.4
WPRO (western Pacific)*	8	11.6	100.0
Total	69	100.0	−

CIS = Counterfeit Incident System; WHO = World Health Organization.

Source: A list of the countries included in each WHO region is available at: http://www.who.int/about/regions/en/.

* Includes Taiwan though the WHO does not officially recognize Taiwan as a member state.
